# Brachyury as a potential modulator of androgen receptor activity and a key player in therapy resistance in prostate cancer

**DOI:** 10.18632/oncotarget.8499

**Published:** 2016-03-31

**Authors:** Filipe Pinto, Nelma Pértega-Gomes, José R. Vizcaíno, Raquel P. Andrade, Flavio M. Cárcano, Rui Manuel Reis

**Affiliations:** ^1^ Life and Health Sciences Research Institute (ICVS), School of Health Sciences, University of Minho, Braga, Portugal; ^2^ ICVS/3B's – PT Government Associate Laboratory, Braga, Portugal; ^3^ Department of Medical Oncology, Dana-Farber Cancer Institute, Harvard Medical School, Boston, Massachusetts, USA; ^4^ Department of Pathology, Centro Hospitalar do Porto, Porto, Portugal; ^5^ CBMR, Centre for Biomedical Research, Universidade do Algarve, Faro, Portugal; ^6^ Regenerative Medicine Program, Department of Medicine and Biomedical Sciences, University of Algarve, Faro, Portugal; ^7^ Clinical Oncology Department, Barretos Cancer Hospital, Barretos, S. Paulo, Brazil; ^8^ Molecular Oncology Research Center, Barretos Cancer Hospital, Barretos, S. Paulo, Brazil

**Keywords:** Brachyury, prostate cancer, therapy resistance

## Abstract

Prostate cancer (PCa) is the most commonly diagnosed neoplasm and the second leading cause of cancer-related deaths in men. Acquisition of resistance to conventional therapy is a major problem for PCa patient management. Several mechanisms have been described to promote therapy resistance in PCa, such as androgen receptor (AR) activation, epithelial-to-mesenchymal transition (EMT), acquisition of stem cell properties and neuroendocrine transdifferentiation (NEtD). Recently, we identified Brachyury as a new biomarker of PCa aggressiveness and poor prognosis. In the present study we aimed to assess the role of Brachyury in PCa therapy resistance. We showed that Brachyury overexpression in prostate cancer cells lines increased resistance to docetaxel and cabazitaxel drugs, whereas Brachyury abrogation induced decrease in therapy resistance. Through ChiP-qPCR assays we further demonstrated that Brachyury is a direct regulator of AR expression as well as of the biomarker AMACR and the mesenchymal markers Snail and Fibronectin. Furthermore, *in vitro* Brachyury was also able to increase EMT and stem properties. By *in silico* analysis, clinically human Brachyury-positive PCa samples were associated with biomarkers of PCa aggressiveness and therapy resistance, including *PTEN* loss, and expression of NEtD markers, *ERG* and *Bcl-2*. Taken together, our results indicate that Brachyury contributes to tumor chemotherapy resistance, constituting an attractive target for advanced PCa patients.

## INTRODUCTION

Prostate cancer (PCa) is the most prevalent malignancy in men and the second leading cause of cancer-related deaths [1]. Despite advances in prevention, early detection, surgical techniques and adjuvant radiotherapy/chemotherapy, progression to advanced prostate cancer and metastasis is a frequent event that hinders patient's cure [2]. The fist-line therapy for PCa patients includes the blockade of androgen receptor (AR) activation and signaling based on androgen-deprivation therapy. This approach is effective at an early phase, but eventually tumors recur, leading to the known metastatic castration-resistant prostate cancer (mCRPC) with a lethal outcome [3]. The exact mechanisms underlying the development of mCRPC are not fully understood. It was proposed that it arises when cancer cells either maintain AR signaling in the absence of normal levels of ligand or no longer require activation of this pathway for survival and proliferation [11]. It has also been suggested an association between chemoresistance and epithelial-to-mesenchymal transition (EMT) in PCa, a mechanism by which cancer cells acquire a higher capacity to invade and further metastasize [13], as well as increased stem cell features (e.g. CD15 and CD133) [14, 15]. Moreover, androgen-deprivation therapy frequently induces the emergence of highly aggressive prostate phenotypes with neuroendocrine (NE) features [expression of chromogranin A (*CHGA*) and synaptophysin (*SYP*)], also called neuroendocrine transdifferentiation (NEtD) [16]. Thus, it is crucial to understand these mechanisms in order to identify therapeutic biomarkers and potential novel therapeutic approaches for these patients. Currently, docetaxel is one of the commonest therapeutic agent given as first-line chemotherapy for patients with mCRPC [4–6]. In the mCRPC patients with docetaxel-resistant phenotypes, it has been shown the survival benefit with second-line therapy using new drugs such as enzalutamide, abiraterone and cabazitaxel [7–10].

The T-box transcription factor Brachyury (T) plays a key role during early embryo gastrulation, a typical EMT process [22]. Lately, Brachyury has also been associated with tumor development and progression [17–21], and its role on EMT, stemness and cancer therapy resistance was described [23–29].

We recently reported Brachyury as a new and independent biomarker of poor prognosis in PCa patients [21, 30]. We also demonstrated that Brachyury could be involved in EMT, stem and neuronal differentiation [21], an indicative of NEtD in PCa. In the present work we aimed to explore whether Brachyury is a molecular driver of the major mechanisms of prostate tumor therapy-resistance, namely AR, EMT, NEtD and stemness, in prostate cancer cells treated with docetaxel and cabazitaxel.

## RESULTS

### Brachyury promotes prostate cancer cell resistance to the chemotherapeutic agents docetaxel and cabazitaxel

**Figure 1:**
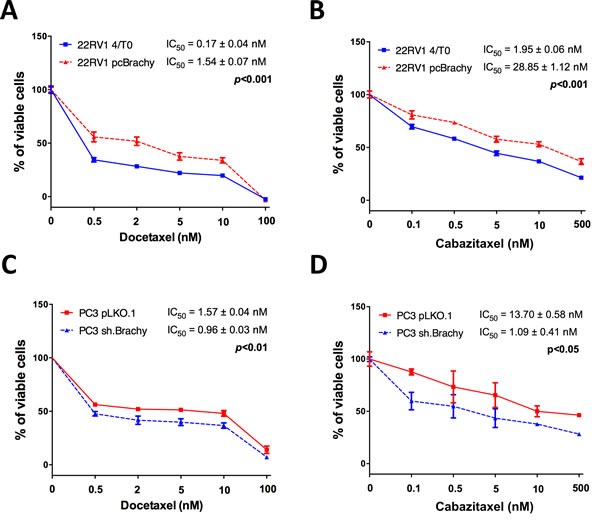
Brachyury promotes resistance to the cytotoxic drugs docetaxel and cabazitaxel Brachyury overexpression increases significantly the IC_50_ of 22RV1 cells for both **A.** docetaxel and **B.** cabazitaxel. Brachyury depletion on PC3 cells decreases the IC_50_ for **C.** docetaxel and **D.** cabazitaxel. Data for drug assays are mean ± S.E.M, of 3 biological independent experiments with 3 technical replicates each.

Comparing only the control cells (transfected with empty vectors), the metastatic PC3 cells (with endogenous Brachyury expression) demonstrated to be more resistant to docetaxel and cabazitaxel than the primary PCa 22RV1 and the bone metastatic DU145 cells (negative for Brachyury expression) (Supplementary Figure S1B). To explore the hypothesis that Brachyury could influence therapy response, we used the primary PCa 22RV1 and the brain metastatic PCa DU145 cell lines (both Brachyury-negative) previously modulated to overexpress Brachyury (pcBrachyury) and the bone metastatic PC3 cell line (Brachyury-positive) with Brachyury sh-mediated depletion (sh.Brachyury) (Supplementary Figure S1A) [21]. Cells were treated with different concentrations of docetaxel or cabazitaxel to determine the IC_50_ of each cell line (22RV1 and DU145 4T/0 *vs* pcBrachyury; PC3 pLKO.1 *vs* sh.Brachyury). All cell lines used are androgen-independent to better explore the implication of Brachyury in castrate resistance prostate cancer (CRPC) therapy. The overexpression of Brachyury in 22RV1 cells was significantly associated with a higher resistance to both cytotoxic drugs [(docetaxel: 4/T0 IC_50_=0.17±0.04nM, pcBrachyury IC_50_=1.54±0.07nM; p<0.001), Figure 1A; (cabazitaxel: 4/T0 IC_50_=1.95±0.06nM, pcBrachyury IC_50_=28.85±1.12nM; p<0.001); Figure 1B]. Similar results were also obtained in the metastatic DU145 cell line with Brachyury overexpression (Supplementary Figure S1C). When Brachyury was depleted on PC3 cells we observed a significant decrease in the IC_50_ for both drugs used, compared with control cells [(docetaxel: pLKO.1 IC_50_=1.57±0.04nM, sh.Brachyury IC_50_=0.96±0.03nM; p<0.01), Figure 1C; (cabazitaxel: pLKO.1 IC_50_=13.37±0.58nM, sh.Brachyury IC_50_=1.09±0.41nM; p<0.05), Figure 1D]. Altogether, these results indicate that the presence of Brachyury in PCa cells is directly associated with resistance to current chemotherapy-mediated treatments used in CRPC therapy.

### Presence of Brachyury in PCa correlates with resistance plasticity mechanisms EMT and stemness

We have previously shown by *in silico* analysis in PCa samples that Brachyury is associated with altered expression of genes involved in epithelial-to-mesenchymal transition (EMT), namely *E* and *N-cadherin*, *Snail*, *TGF-β1*, *fibronectin*, *MMP14* and *MMP24*, and in stemness (CD44) [21]. To corroborate our previous results, we performed RT-qPCR in a primary PCa cell line (22RV1) with modulated Brachyury expression (Supplementary Figure S2A). We found that Brachyury overexpression was significantly associated with the decrease of the epithelial marker *E-cadherin* and an increased expression of the mesenchymal marker *Snail* (Supplementary Figure S2A).

**Figure 2:**
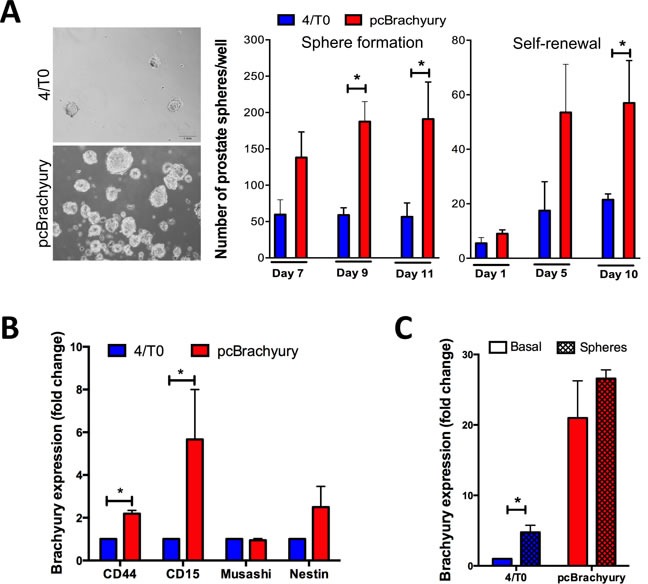
Brachyury promotes stem cell properties in PCa cells and is re-expressed under pressure conditions **A.** In 22RV1 cells Brachyury is able to enhance the capacity to form prostate-spheres and self-renewal over time, as well as **B.** increases the expression of stem cell markers *CD44* and *CD15*. **C.** Brachyury is up-regulated in negative 22RV1 cells when cultured under stem cell medium. Results of prostate-sphere are representative of two biological independent experiments in duplicate. Graphs of expression are mean ± S.E.M of 3 biological independent experiments with 3 technical replicates each. *, *p* < 0.05.

We further evaluated the involvement of Brachyury in gain of stemness properties in PCa cells. We found that Brachyury is able to increase the number of prostate-spheres and the self-renewal capacity over time in 22RV1 cells Brachyury-positive cells (pcBrachyury) compared with Brachyury-negative cells (4/T0) (Figure 2A). Moreover, Brachyury was able to significantly increase the expression of the stem markers *CD44* and *CD15* (Figure 2B). Interestingly, we observed that 22RV1 PCa cells under sphere-forming conditions showed an increase of *Brachyury* expression, when compared with cells cultured in basal/monolayer conditions, with statistical significance in endogenous negative cells (4/T0) (Figure 2C). The influence of Brachyury in stemness was also addressed in metastatic DU145 cell line with ectopic Brachyury expression and in PC3 cell line with Brachyury depletion. Although we have found an increased number of aggregates in Brachyury-positive cells, they not form spheres (Supplementary Figure S2B).

Together, Brachyury overexpression occurring in PCa tissues could contribute to tumor cell plasticity mechanisms as EMT and gain of stem cell properties. Moreover, we showed that prostate tumor cells could up-regulate Brachyury under stemness conditions.

### Brachyury is co-expressed with AR, ERG, Bcl-2, NEtD markers and inversely with PTEN in human PCa tissues

**Figure 3:**
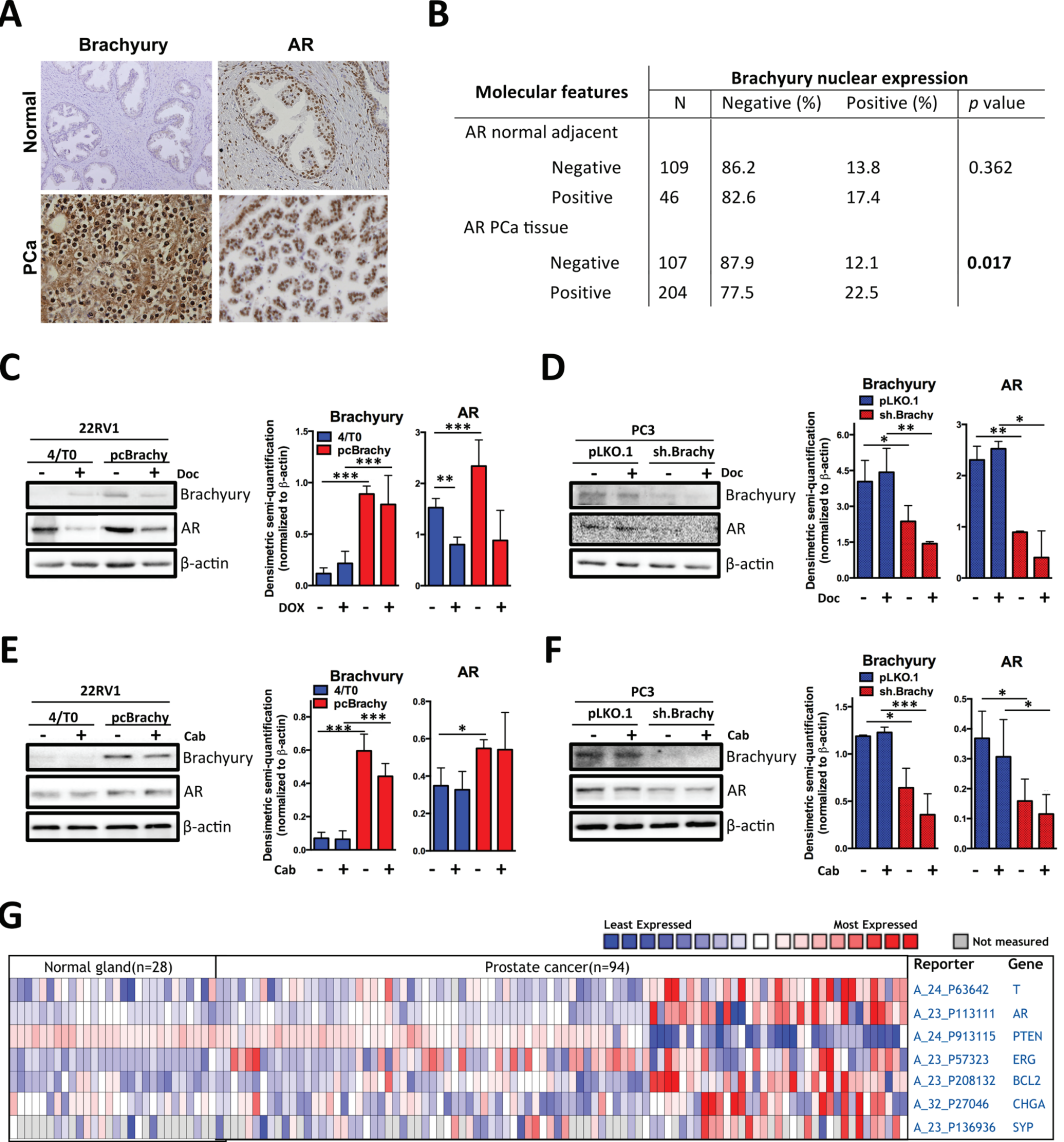
Brachyury is associated with AR, Bcl-2, ERG, CHGA and SYP expression and with loss of PTEN expression **A.** Representative images of Brachyury and AR staining in normal and primary PCa tissues. **B.** Correlation of Brachyury and AR staining in prostate tissues. Nuclear Brachyury staining is correlated with AR only in primary PCa tissues. **C.**-**F.** Western blot analyses for Brachyury and AR expression in prostate cell lines treated with docetaxel (Doc) or with cabazitaxel (Cab). Graphs represent the densimetric semi-quantification of western blot images. **G.** Heatmap showing the co-expression of *Brachyruy* (*T*), *AR*, *PTEN*, *Bcl-2*, *ERG*, *CHGA* and *SYP* in normal and PCa human samples from Oncomine database.

To assess whether Brachyury is related with AR expression, we analyzed 155 normal prostate tissues and 311 primary PCa tissues by immunohistochemistry (Figure 3A and 3B), previously characterized for Brachyury expression [21]. We found that presence of Brachyury protein in the nucleus of primary PCa tissues is statistically associated with the presence of AR (p=0.017; Figure 3B), a feature not observed in normal prostate tissues (*p*=0.362; Figure 3B). Moreover, western blot analyses of PCa cell lines with Brachyury modulation showed that Brachyury overexpression significantly increases AR expression and when Brachyury is depleted there is a concomitant decrease of AR expression (Figure 3C, 3D, 3E, 3F). We also observed that docetaxel treatment is able to decrease AR expression in 22RV1 cells in absence of Brachyury (4/T0 22RV1, p<0.05), but not in the Brachyury-overexpressing cells. No effect was observed in PC3 cell line, indicating that effect of docetaxel in AR downregulation is cell-type dependent (Figure 3C, 3D, 3E, 3F). Based on these results, we may hypothesize that increased AR expression promoted by Brachyury can lead to docetaxel and cabazitaxel resistance (Figure 1).

The correlation between Brachyury and AR was also corroborated by *in silico* analysis in a series of 28 non-tumoral and 94 PCa samples (Figure 3G). We found that expression of the *AR* was significantly increased in PCa tissues as compared to the normal prostate gland and is co-expressed with *Brachyury* (*T*) (Figure 3A). We extended our *in silico* analysis to other genes described to be associated with poor patient prognosis and with PCa progression to a castration-resistant phenotype [31, 32]. *Brachyury* co-expression was correlated with *PTEN* (the most frequently downregulated tumor-suppressor gene in PCa), *Bcl-2* (associated with therapy resistance) and *ERG* (overexpressed due *TMPRSS2-ERG* fusion). As shown in Figure 3G, a strong association was found between *Brachyury* expression and *Bcl-2* and *ERG expression* and *PTEN* loss (Figure 3G).

We previously showed that genes co-expressed with Brachyury are clustered in processes of neuron differentiation and central nervous system development [21]. This finding is indicative that Brachyury could be involved in the NEtD process. To better understand this mechanism we investigated the co-expression of *Brachyury* with *chromogranin A* (*CHGA*) and *synaptophysin* (*SYP) in silico*, two NEtD markers described to distinguish prostate tumors with NE features. We found that *Brachyury* is strongly correlated with NEtD markers, reinforcing its involvement in NEtD (Figure 3G). A gene signature that includes expression of *Brachyury*, *CHGA, SYP, AR* and loss of *PTEN* was found (Figure 3G), being correlated with more aggressive tumors. The direct involvement of *Brachyury* in *CHGA*, *SYP*, *AR, Bcl2, ERG* and *PTEN* expression is still unclear; however, the association with these well-established markers of PCa progression supports the importance of Brachyury as a new biomarker of PCa aggressiveness.

### Brachyury directly binds to the promoters of Snail and Fibronectin (mesenchymal biomarkers), AMACR (PCa biomarker) and AR

**Figure 4:**
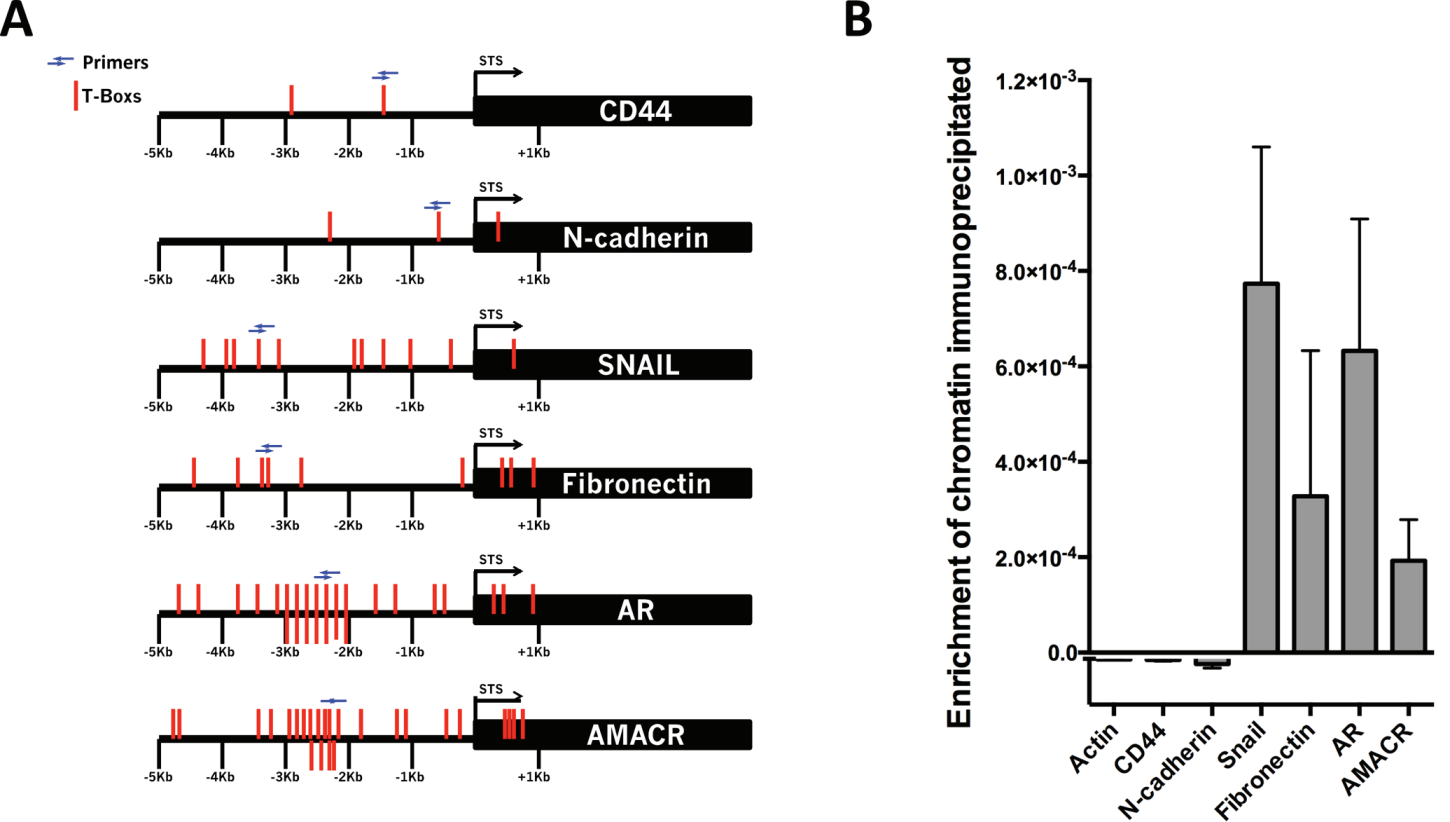
Brachyury directly binds to the promoter regions of Snail, Fibronectin, AMACR and AR **A.** Squematic representation of the T-Boxs (red) in the region analyzed (6Kbs) and the localization of the primers (blue arrows) used in the ChIP-qPCR. **B.** Brachyury showed an enrichment of chromatin immunoprecipitated for *Snail, Fibronectin, AMACR* and *AR*, but not for the negative control *β-actin*, *CD44* and *N-cadherin*. The graph represents mean ± S.E.M. of three biological experiments with 3 technical replicates.

A correlation between the expression of *Brachyury* with CD44, *N-cadherin, Snail, fibronectin*, AMACR and AR (present study and [21]) in PCa cells suggested that Brachyury could directly regulate these genes. To validate this hypothesis, we performed ChIP-qPCR in PC3 cell line with endogenous Brachyury expression. Analyses of 6Kbs (−5Kb to +1Kb) of the *CD44*, *N-cadherin*, *Snail, Fibronectin, AMACR* and *AR* promoter regions revealed presence of T-Box binding sites in all genes (Figure 4A, T-Boxs represented in red). However, a higher number T-Box binding site clusters was only found on *AR* (26 T-Boxs), *AMACR* (25 T-Boxs), *Snail* (11 T-Boxs) and Fibronectin (9 T-Boxs) in opposite with *CD44* and *N-cadherin* that only present 2 and 3 T-Box sites, respectively (Figure 4A).

The cross-linked DNA-protein complexes were immunoprecipitated with anti-Brachyury or with control IgG antibody. PCR amplification was performed using primers flanking regions with T-Boxs (blue arrows, Figure 4A). The results of ChIP-qPCR analysis showed that upstream regions of the mesenchymal marker genes *Snail* and Fibronectin, the PCa biomarker *AMACR* and *AR* precipitated with anti-Brachyury antibody showing that Brachyury directly binds to regulatory elements of these genes and suggests possibly regulation of those genes (Figure 4B).

## DISCUSSION

In the present study we provided the first evidences that Brachyury regulates several biological mechanisms associated with of PCa therapy resistance. Importantly, we showed that Brachyury directly regulates AR expression and promotes chemoresistance in PCa cells. Moreover, we also showed that Brachyury might also be a direct regulator of AMACR biomarker, in agreement with our previously results [21]. These results indicate that Brachyury alone or in combination with AMACR, could be used in routine setting as a PCa diagnosis biomarker.

Using ChiP-qPCR we were able to show that Brachyury is a direct regulator of AR. This finding was further validated by immunohistochemistry of a cohort of PCa patients and *in silico* analysis. We found a particular association when Brachyury is present only in the nucleus, not in the cytosol. We believe that correlation of Brachyury and AR in the nucleus could be a direct consequence of Brachyury role in activation *AR* expression and consequently therapy resistance. Moreover, Brachyury presence in the cytosol could be a surrogate marker of PCa aggressiveness. By ChiP-qPCR, we also observed that *Snail* and *Fibronectin -* typical mesenchymal markers - are directly regulated by Brachyury, strongly suggesting its role in the EMT process. These results are in agreement with our previous study, where we showed that Brachyury increases migration and invasion capabilities in PCa cells and it was correlated with EMT markers (*fibronectin*, *MMP14*, *MMP24*, *Snail*, *IL8* and *TGF-β1*) by *in silico* analysis [21]. Moreover, Brachyury overexpression increases expression of stem cell markers (*CD44*, *CD15* and *Nestin*) being associated with increased capacity of PCa cells to form prostate-spheres and with self-renewal in 22RV1 cell line. The absence of effect on stem in the other PCa cell lines suggests that Brachyury is not the only mechanism responsible for increased stem cell capabilities, but probably it involve more complex mechanisms, once the genetic background of each cell line could influence it. Furthermore, Brachyury was also associated with NE markers (*CHGA* and *SYP*), reinforcing the hypothesis that it is involved in the NEtD process and consequently higher aggressive tumors. Brachyury was previously associated with the NE marker CHGA, in colorectal cancer, distinguishing a specific subpopulation of stem cells [33].

The involvement of Brachyury in tumor resistance, namely to docetaxel, was previously described in breast cancer [27, 28]. In accordance, we demonstrated that Brachyury promotes higher resistance not only to docetaxel, but also to cabazitaxel, two currently chemotherapeutic agents used in CRPC treatment. A recent genome-wide analysis on *AR* that showed an enrichment of T-Box motifs in LAPC-4 prostate cells after chemotherapy [34], indicating that Brachyury is involved in *AR* expression during PCa therapy and consequently increased therapy resistance. We hypothesize that this effect on Brachyury expression can be extrapolated to other pressure conditions, as longer periods exposed to chemotherapy. Interestingly, we found that Brachyury can be re-expressed under pressure conditions, as can happen during chemotherapy of PCa. Indeed, lung cancer cells exposed to successive cycles of chemotherapy (docetaxel) selection and recovery showed elevated Brachyury expression levels compared with untreated cells [18].

Several molecular events, such as *TMPRSS2-ERG* fusion, *Bcl-2* overexpression, as well as *PTEN* loss has been described as major biomarkers of PCa aggressiveness and therapy response [35–38]. Herein, we showed that *Brachyury* is co-expressed with the ETS transcription factor *ERG*, *Bcl-2* and inversely co-expressed with *PTEN*. In hepatocellular carcinoma cells, Brachyury showed to increase Akt signaling activation by inhibition of PTEN expression [39]. This recent finding together with the present data, suggest that in PCa Brachyury can suppress PTEN leading to increased activation of Akt signaling and consequently aberrant transcription of down-stream checkpoint genes, like *Bcl-2* and *ERG*. Aberrant expression of an ETS transcription factor (usually ERG) is also found in response to the activated AR [38], as those promoted by Brachyury overexpression. This signaling crosstalk could provide the selective advantage at the cellular level to allow PCa progress and be resistant to current therapy. Therefore, Brachyury can constitute an attractive therapeutic target.

Recently a Phase II clinical trial was initiated with the yeast-Brachyury vaccine GI-6301 (www.clinicaltrials.gov, NCT02383498, 2015) to treat patients with advanced carcinomas. Preliminary results showed that Brachyury vaccine was well tolerated and Brachyury-specific CD8+ and/or CD4+ T-cell responses were present in the blood of some patients post- versus pre-vaccination [40, 41]. This evidence together with results obtained in the present study indicates that *Brachyury* could be used as an immunotherapeutic target in patients with advanced PCa.

In conclusion, we have demonstrated the importance of Brachyury in PCa resistance to chemotherapeutic agents by modulating several tumorigenic and aggressive mechanisms. Additionally the previous association of Brachyury with PCa patient prognosis [21] and current therapy response suggest that Brachyury could be a important theranostic biomarker for advanced prostate cancer patients.

## MATERIALS AND METHODS

### Brachyury and androgen receptor (AR) correlation in human prostate tissues

AR and Brachyury co-expression was assessed in a cohort of 155 normal prostate tissues and 311 primary PCa tissues, where we previously described the expression and distribution of Brachyury [21].

For AR staining histological slides with 4 μm-thick tissue sections were subjected to immunohistochemistry analysis according to the streptavidin-biotin peroxidase complex system (UltraVision Large Volume Detection System Anti-Polyvalent, HRP; LabVision Corporation), using the primary antibody raised against AR (diluted 1:750; sc-816, Santa Cruz Biotechnology, Inc). AR immunohistochemistry results were semi-quantitatively scored. Tumors were graded as negative when expression was seen in less than 5% of nuclei, 1 when staining 5% to 10% of nuclei, 2 when staining more than 10% to 50% of nuclei, and 3 when staining more than 50% of nuclei. Samples with scores 0, 1 were considered negative and those with scores 2 and 3 were considered positive.

### Cell lines and cell culture conditions

Human prostate cell lines 22RV1, DU145 and PC3 (ATCC-American Type Culture Collection, MD, USA) were grown in Roswell Park Memorial Institute medium (RPMI)-1640 (GIBCO®, Invitrogen) cell culture medium supplemented with 10% Fetal Bovine Serum (FBS) (GIBCO®, Invitrogen) and 1% Penicillin-Streptomycin (P/S) (GIBCO®, Invitrogen).

### Brachyury over-expression and silencing in PCa cells

22RV1 and DU145 were transfected with full-length human Brachyury cDNA in pcDNA4/T0 vector (designed as pcBrachyury) or with empty vector (4/T0). For Brachyury silencing, PC3 cells were transfected with pLKO.1 plasmid containing Brachyury-specific shRNA (sh.Brachyury) or empty vector alone (pLKO.1) [21]. Transfected 22RV1 and DU145 cells were maintained under selection with Zeocin 50μg/ml (Invitrogen). Stable PC3 cells with depleted endogenous Brachyury expression were maintained with 5μg/ml of puromycin (Sigma Aldrich), as previously described [21].

### Western blot analysis

Cells were lysed in 50 mM Tris pH 7.6-8, 150 mM NaCl, 5 mM EDTA, 1 mM Na_3_VO_4_, 10 mM NaF, 10 mM NaPyrophosphate, 1% NP-40 and 1/7 of protease cocktail inhibitors (Roche). Proteins were resolved on standard 12% SDS-PAGE gel and transferred onto nitrocellulose membranes. The immunodetection was achieved using antibodies for human Brachyury (AF2085, R&D Systems), AR (ab108341, Abcam) and β-actin (sc-1616, Santa Cruz Biotechnology). Detection was done by chemiluminescence (SuperSignal West Femto Chemiluminescent Substrate; Thermo Scientific). Protein expression quantification was performed by ImageJ software (version 1.44).

### RNA extraction and reverse-transcriptase (RT) cDNA synthesis

Total RNA was extracted from cell lines using TRIzol® Reagent (Invitrogen). The concentration and purity of RNA was evaluated by spectrophotometry (Nanodrop Technologies, Inc). One μg of RNA was used for cDNA synthesis, using the High-Capacity cDNA Reverse Transcription Kit (Applied Biosystems), as recommended by the manufacturer.

### Quantitative polymerase chain reaction (qPCR)

qPCR was performed in a CFX96 detection system (BioRad), using SSOfast Evagreen supermix (BioRad) following the manufacturer's instructions. Optimized cycling conditions for qPCR was as follows: enzyme activation for 30 seconds at 95°C; 45 cycles of denaturation at 95°C for 5 seconds and annealing/extension at 59°C for 5 seconds. Melting curve was assessed at 65°C – 95°C (in 0.5°C increase) at each 5 seconds/step. The primers used are presented in Supplementary Table 1. The expression levels were normalized to β-actin by ΔCT (relative expression) or by ΔΔCT method (fold-change). Results are presented as the mean ± SD of 3 independent experiences in triplicate.

### T- Box binding sites and primer design

A total of 6Kbs (−5KB to +1Kb) for *CD44*, *Snail*, *Fibronectin*, *N-cadherin, AMACR* and *AR* were screened for T-Box binding consensus sequences (Supplementary Figure S3A). β-actin was used as negative control of Brachyury binding (data from ChIP-sequencing in human chordoma cells) [42]. To look for T-Box binding sites we used JASPAR database (http://jaspar.genereg.net) that contains a matrix-based nucleotide profile of binding preference for transcription factors from multiple species. Primers (Supplementary Table 1) were designed flanking regions enriched in T-Box binding sites.

### Chromatin immunoprecipitation (ChIP) – qPCR

ChIP-qPCR analysis was performed in PC3 cell line containing Brachyury endogenous expression. The protocol used was adapted from [42]. Briefly, adherent PC3 cells (~3×10^7^) were fixed for 5 minutes *in situ* with 1.27% formaldehyde solution (Sigma F8775). Immediate quenching of formaldehyde with glycine 2.5M (1/20 of total volume) was performed for 5 minutes. Cells were washed twice with 1× Dulbecco's phosphate buffered saline (PBS 1X), harvested using a cell scraper (Sarstedt, NC, USA), centrifuged and the pellet cells were quickly washed with ice-cold PBS 1X. PBS was totally removed and pellets were snap-frozen in liquid nitrogen and stored at −80°C.

Pellets were homogenized with cell lysis buffer (10mM Tris-HCl pH=7.5, 10mM NaCl, 0.5% NP-40) and left on ice for 1-2 hours. After centrifugation, supernatant pellet was re-suspended in nuclei lysis buffer (50mM Tris-HCl pH=7.5, 10mM EDTA pH=8.0, 1% SDS) and kept on ice for 15-30 minutes. At this stage, 2X volumes of IP dilution buffer (16.7mM Tris-HCl pH=7.5, 167mM NaCl, 1.2mM EDTA pH=8.0, 0.01% SDS) was added to each pellet of cells. Chromatin was solubilized and sheared to fragments of 100-500bp peaking around 200bp (Supplementary Figure S3B) using an ultrasonic processor (Vibra-cell sonicator; Sonics and Materials, INC) (10 cycles of 30 seconds shock waves at 3 Watt interrupted by 30 seconds pauses).

Magnetic Dynabeads Protein G (Novex, Life Technologies) were blocked in PBS containing 0.5% (w/v) BSA and coupled to ChIP-grade antibody (or control rabbit IgG) at 4°C overnight. 6μg of Brachyury antibody (sc-20109, Santa Cruz Biotechnology) was coupled to 50μl of the appropriate Dynabeads Protein G. The soluble nuclear extract was prepared for immunoprecipitation by adding 80μl of 10% Triton X-100 per 1ml of chromatin. A sample of 50μl of ChIP input was collected at this stage and stored at 4°C. Then the nuclear extract was combined with antibody-coupled beads (Brachyury or control IgG) and incubated at 4°C overnight with rotation. After incubation, magnetic beads were precipitated using an eppendorf-magnet and washed with pre-chilled RIPA buffer (50mM HEPES pH=7.6, 500mM LiCl, 1mM EDTA, 1% NP-40, 0.7% (w/v) Na-deoxycholate). Bound chromatin was eluted from the beads with elution buffer (50mM Tris-HCl pH=8.0, 10mM EDTA pH=8.0, 1% SDS) and reversed cross-linked in a thermomixer (65°C, 6-hours). After reverse cross-link, 1 volume of TE pH=8.0 and 200μg/ml of RNase A (Invitrogen) was added to the samples for a 1-hour incubation at 37°C. The samples were further treated with 200μg/ml of proteinase K (Ambion) for 1-hour at 55°C. Finally, the DNA fragments were purified with phenol:chloroform:isoamylalcohol (phenol:Chl:1A, Sigma) and precipitated in 1/20 volume of 5M NaCl, 20μg of glycogen and ethanol. ChIP-chromatin was diluted in 30μl of DNase-free water.

DNA enrichment was quantified in real-time PCR relative to locus-specific standard curves using CFX96TM system (BioRad). The percentage of DNA enrichment for a specific antibody was normalized to the amount of total DNA (equivalent to Input DNA): (Ab-IgG)/Input. Real-time PCR was carried out with the following conditions: 30 seconds at 95°C, 5 seconds at 59°C and 72°C respectively, 60 cycles. qPCR runs were performed with 3 technical replicates.

### Prostate-sphere assay

Prostate sphere assay (adapted from [43]) was used to evaluate the capacity of prostate cells to grow and form spheres in suspension, an indicative of higher stemness phenotype. Each sample of cells was counted (1×10^3^cells/well), resuspended in 2mL of Neurobasal media (Invitrogen, Carlsbad, CA), supplemented with 2 mM glutamine and B27 (Invitrogen) and plated on a 12-well plate. Spheres were counted 3–15 days after plating. For passaging of spheres, media was collected centrifuged at 900rpm for 5 minutes to pellet the spheres. The supernatant was then removed and spheres were mechanically dissociated in 1mL of fresh medium to obtain single cell suspension. Cells were counted and re-plated at a density of 1×10^3^cells/well to evaluate the capacity of self-renewal.

### Drug treatment and determination of half-maximal inhibitory concentration (IC_50_)

The conventional and metastatic-second line PCa cytotoxic drugs docetaxel and cabazitaxel (MedChemTronica), respectively, were used to study the therapeutic value of Brachyury on PCa cells. Drugs were diluted in dimethyl sulfoxide (DMSO), as recommended by the manufacture. To determine the IC_50_ of the drugs 4×10^3^ cells/well for 22RV1 and 2×10^3^ cells/well for PC3 were plated into 96-well plates in triplicate and allowed to adhere overnight. After 6-hours of starvation (RPMI only) cells were treated with different concentrations of docetaxel (0, 0.5, 2, 5, 10, 100 and 1000nM) or cabazitaxel (0, 0.1, 0.5, 5, 10, 500 and 1000nM) in culture medium supplemented with 10% FBS. After 48-hours of drug incubation the cellular viability was assessed by MTS (Promega, USA) in a 10:1 ratio and incubated in a humidified atmosphere at 37°C and 5% CO_2_. Following 2 hours of incubation the optical density was determined at 490nm in the Varioscan-Flash plate reader (Thermo Scientific). The cells treated with vehicle were incubated with culture medium containing 10% FBS and 1% DMSO. To assess AR and Brachyury protein expression after drug treatment cells were cultured (3×10^5^ cells/well) in a 6-well plate and allowed to adhere over-night. In the next day cells were treated with docetaxel or cabazitaxel (lowest IC_50_ for each cell line was used) for 24 hours. Controls were treated with 1% DMSO. Cells were then collected for protein extraction and western blot analyses.

### *In silico* microarray expression analysis

Analysis of co-expression of *Brachyury*, *AR, PTEN, ERG, Bcl-2* and genes involved in NEtD (*SYP* and *CHGA*) was performed using data extracted from Oncomine database [44, 45]. For this study we used the Grasso dataset [46] compromising 28 normal prostate glands and 94 PCa tissues, the only dataset with the information for the 7 genes of interest.

### Statistical analysis

Correlations between Brachyury and AR expression in human samples were performed using the chi-square test (χ2-test). Simple comparisons between two different conditions were analyzed using Student's t test. The statistical analysis was performed using SPSS software (version 19.0) or using Prism GraphPad software (version 5.0a). The level of significance in the statistical analyses is indicated as *=p<0.05, **=p<0.01 or ***=p<0.001.

## SUPPLEMENTARY MATERIAL TABLE AND FIGURES



## References

[1] Siegel R, Ma J, Zou Z, Jemal A (2014). Cancer statistics, 2014. CA Cancer J Clin.

[2] van Dodewaard-de Jong JM, Verheul HM, Bloemendal HJ, de Klerk JM, Carducci MA, van den Eertwegh AJ (2015). New Treatment Options for Patients With Metastatic Prostate Cancer: What Is The Optimal Sequence?. Clin Genitourin Cancer.

[3] Moul JW, Evans CP, Gomella LG, Roach M, Dreicer R (2011). Traditional approaches to androgen deprivation therapy. Urology.

[4] Tannock IF, de Wit R, Berry WR, Horti J, Pluzanska A, Chi KN, Oudard S, Théodore C, James ND, Turesson I, Rosenthal MA, Eisenberger MA, TAX 327 Investigators (2004). Docetaxel plus prednisone or mitoxantrone plus prednisone for advanced prostate cancer. NEJM.

[5] Berthold DR, Pond GR, Soban F, de Wit R, Eisenberger M, Tannock IF (2008). Docetaxel plus prednisone or mitoxantrone plus prednisone for advanced prostate cancer: updated survival in the TAX 327 study. J Clin Oncol.

[6] Sweeney CJ, Chen YH, Carducci M, Liu G, Jarrard DF, Eisenberger M, Wong YN, Hahn N, Kohli M, Cooney MM, Dreicer R, Vogelzang NJ, Picus J (2015). Chemohormonal Therapy in Metastatic Hormone-Sensitive Prostate Cancer. NEJM.

[7] Scher HI, Fizazi K, Saad F, Taplin ME, Sternberg CN, Miller K, de Wit R, Mulders P, Chi KN, Shore ND, Armstrong AJ, Flaig TW, Fléchon A, Mainwaring P, Fleming M, Hainsworth JD (2012). Increased survival with enzalutamide in prostate cancer after chemotherapy. NEJM.

[8] de Bono JS, Logothetis CJ, Molina A, Fizazi K, North S, Chu L, Chi KN, Jones RJ, Goodman OB, Saad F, Staffurth JN, Mainwaring P, Harland S, Flaig TW, Hutson TE, Cheng T (2011). Abiraterone and increased survival in metastatic prostate cancer. NEJM.

[9] de Bono JS, Oudard S, Ozguroglu M, Hansen S, Machiels JP, Kocak I, Gravis G, Bodrogi I, Mackenzie MJ, Shen L, Roessner M, Gupta S, Sartor AO, TROPIC Investigators (2010). Prednisone plus cabazitaxel or mitoxantrone for metastatic castration-resistant prostate cancer progressing after docetaxel treatment: a randomised open-label trial. Lancet.

[10] Oudard S (2011). TROPIC: Phase III trial of cabazitaxel for the treatment of metastatic castration-resistant prostate cancer. Future Oncol.

[11] Lamont KR, Tindall DJ (2011). Minireview: Alternative activation pathways for the androgen receptor in prostate cancer. Mol Endocrinol.

[12] Nouri M, Ratther E, Stylianou N, Nelson CC, Hollier BG, Williams ED (2014). Androgen-targeted therapy-induced epithelial mesenchymal plasticity and neuroendocrine transdifferentiation in prostate cancer: an opportunity for intervention. Front Oncol.

[13] Sun Y, Wang BE, Leong KG, Yue P, Li L, Jhunjhunwala S, Chen D, Seo K, Modrusan Z, Gao WQ, Settleman J, Johnson L (2012). Androgen deprivation causes epithelial-mesenchymal transition in the prostate: implications for androgen-deprivation therapy. Cancer Res.

[14] Tang DG, Patrawala L, Calhoun T, Bhatia B, Choy G, Schneider-Broussard R, Jeter C (2007). Prostate cancer stem/progenitor cells: Identification, characterization, and implications. Mol Carcinog.

[15] Xiao W, Graham PH, Power CA, Hao J, Kearsley JH, Li Y (2012). CD44 is a biomarker associated with human prostate cancer radiation sensitivity. Clin Exp Metastasis.

[16] Yang JC, Ok JH, Busby JE, Borowsky AD, Kung HJ, Evans CP (2009). Aberrant activation of androgen receptor in a new neuropeptide-autocrine model of androgen-insensitive prostate cancer. Cancer Res.

[17] Imajyo I, Sugiura T, Kobayashi Y, Shimoda M, Ishii K, Akimoto N, Yoshihama N, Kobayashi I, Mori Y (2012). T-box transcription factor Brachyury expression is correlated with epithelial-mesenchymal transition and lymph node metastasis in oral squamous cell carcinoma. Int J Oncol.

[18] Roselli M, Fernando RI, Guadagni F, Spila A, Alessandroni J, Palmirotta R, Costarelli L, Litzinger M, Hamilton D, Huang B, Tucker J, Tsang KY, Schlom J, Palena C (2012). Brachyury, a driver of the epithelial-mesenchymal transition, is overexpressed in human lung tumors: an opportunity for novel interventions against lung cancer. Clin Cancer Res.

[19] Fernando RI, Litzinger M, Trono P, Hamilton DH, Schlom J, Palena C (2010). The T-box transcription factor Brachyury promotes epithelial-mesenchymal transition in human tumor cells. J Clin Invest.

[20] Pinto F, Campanella NC, Abrahão-Machado LF, Scapulatempo-Neto C, de Oliveira AT, Brito MJ, Andrade RP, Guimarães DP, Reis RM (2016). The embryonic Brachyury transcription factor is a novel biomarker of GIST aggressiveness and poor survival. Gastric Cancer.

[21] Pinto F, Pertega-Gomes N, Pereira MS, Vizcaino JR, Monteiro P, Henrique RM, Baltazar F, Andrade RP, Reis RM (2014). T-box transcription factor Brachyury is associated with prostate cancer progression and aggressiveness. Clin Cancer Res.

[22] Hotta K, Takahashi H, Satoh N, Gojobori T (2008). Brachyury-downstream gene sets in a chordate, Ciona intestinalis: integrating notochord specification, morphogenesis and chordate evolution. Evol Dev.

[23] Fernando RI, Castillo MD, Litzinger M, Hamilton DH, Palena C (2011). IL-8 signaling plays a critical role in the epithelial-mesenchymal transition of human carcinoma cells. Cancer Res.

[24] Imajyo I, Sugiura T, Kobayashi Y, Shimoda M, Ishii K, Akimoto N, Yoshihama N, Kobayashi I, Mori Y (2012). T-box transcription factor Brachyury expression is correlated with epithelial-mesenchymal transition and lymph node metastasis in oral squamous cell carcinoma. Int J Oncol.

[25] Shimoda M, Sugiura T, Imajyo I, Ishii K, Chigita S, Seki K, Kobayashi Y, Shirasuna K (2012). The Tbox transcription factor Brachyury regulates epithelial-mesenchymal transition in association with cancer stem-like cells in adenoid cystic carcinoma cells. BMC Cancer.

[26] Jezkova J, Williams JS, Jones-Hutchins F, Sammut SJ, Gollins S, Cree I, Coupland S, McFarlane RJ, Wakeman JA (2014). Brachyury regulates proliferation of cancer cells via a p27Kip1-dependent pathway. Oncotarget.

[27] Palena C, Roselli M, Litzinger MT, Ferroni P, Costarelli L, Spila A, Cavaliere F, Huang B, Fernando RI, Hamilton DH, Jochems C, Tsang KY, Cheng Q, Lyerly HK, Schlom J, Guadagni F (2014). Overexpression of the EMT driver brachyury in breast carcinomas: association with poor prognosis. J Natl Cancer Inst.

[28] Huang B, Cohen JR, Fernando RI, Hamilton DH, Litzinger MT, Hodge JW, Palena C (2013). The embryonic transcription factor Brachyury blocks cell cycle progression and mediates tumor resistance to conventional antitumor therapies. Cell Death Dis.

[29] Sarkar D, Shields B, Davies ML, Müller J, Wakeman JA (2012). BRACHYURY confers cancer stem cell characteristics on colorectal cancer cells. Int J Cancer.

[30] Thomas C (2014). Prostate cancer: Brachyury--a biomarker for progression and prognosis?. Nat Rev Urol.

[31] Yoshimoto M, Cunha IW, Coudry RA, Fonseca FP, Torres CH, Soares FA (2007). Squire JA FISH analysis of 107 prostate cancers shows that PTEN genomic deletion is associated with poor clinical outcome. Br J Cancer.

[32] Trotman LC, Niki M, Dotan ZA, Koutcher JA, Di Cristofano A, Xiao A, Khoo AS, Roy-Burman P, Greenberg NM, Van Dyke T, Cordon-Cardo C, Pandolfi PP (2003). Pten dose dictates cancer progression in the prostate. PLoS Biol.

[33] Jezkova J, William JS, Pinto F, Sammut SJ, Wiliams GT, Gollins S, McFarlane RJ, Reis RM, Wakeman JA (2016). Brachyury identifies a class of enteroendocrine cells in normal human intestinal crypts and colorectal cancer. Oncotarget.

[34] Perets R, Kaplan T, Stein I, Hidas G, Tayeb S, Avraham E, Ben-Neriah Y, Simon I, Pikarsky E (2012). Genome-wide analysis of androgen receptor targets reveals COUP-TF1 as a novel player in human prostate cancer. PLoS One.

[35] Huang H, Cheville JC, Pan Y, Roche PC, Schmidt LJ, Tindall DJ (2001). PTEN induces chemosensitivity in PTEN-mutated prostate cancer cells by suppression of Bcl-2 expression. J Biol Chem.

[36] Fallahabadi ZR, Noori Daloii MR, Mahdian R, Behjati F, Shokrgozar MA, Abolhasani M, Asgari M, Shahrokh H (2016). Frequency of PTEN alterations, TMPRSS2-ERG fusion and their association in prostate cancer. Gene.

[37] Tamaki H, Harashima N, Hiraki M, Arichi N, Nishimura N, Shiina H, Naora K, Harada M (2014). Bcl-2 family inhibition sensitizes human prostate cancer cells to docetaxel and promotes unexpected apoptosis under caspase-9 inhibition. Oncotarget.

[38] Squire JA (2009). TMPRSS2-ERG and PTEN loss in prostate cancer. Nat Genet.

[39] Du R, Wu S, Lv X, Fang H, Wu S, Kang J (2014). Overexpression of brachyury contributes to tumor metastasis by inducing epithelial-mesenchymal transition in hepatocellular carcinoma. J Exp Clin Cancer Res.

[40] Heery CR, Singh H, Marte JL, Madan RA, O'sullivan Coyne GH, Farsaci, Rodell TC, Palena C, Scholm J, Gulley JL (2014). NCI experience using yeast-brachyry vaccine (GI-6301) in patients with advanced chordoma. J Clinical Oncol.

[41] Shing H, Heery CR, Marte JL, Farsaci B, Madan RA, O'sullivan Coyne GH, Farsaci, Rodell TC, Palena C, Scholm J, Gulley JL (2014). A phase I study of a yeast-based therapeutic cancer vaccine, GI-6301, targeting brachyury in patients with metastatic carcinoma. J Clinical Oncol.

[42] Nelson AC, Pillay N, Henderson S, Presneau N, Tirabosco R, Halai D, Berisha F, Flicek P, Stemple DL, Stern CD, Wardle FC, Flanagan AM (2012). An integrated functional genomics approach identifies the regulatory network directed by brachyury (T) in chordoma. J Pathol.

[43] Lang SH, Anderson E, Fordham R, Collins AT (2010). Modeling the Prostate Stem Cell Niche: An Evaluation of Stem Cell Survival and Expansion *In Vitro*. Stem Cells Dev.

[44] Oncomine Ann Arbor, MI:Compendia Bioscience@ 2007–2010. https://www.oncomine.org/.

[45] Rhodes DR, Kalyana-Sundaram S, Mahavisno V, Varambally R, Yu J, Briggs BB, Barrette TR, Anstet MJ, Kincead-Beal C, Kulkarni P, Varambally S, Ghosh D, Chinnaiyan AM (2007). Oncomine 3. 0: Genes, pathways, and networks in a collection of 18,000 cancer gene expression profiles. Neoplasia.

[46] Grasso CS, Wu YM, Robinson DR, Cao X, Dhanasekaran SM, Khan AP, Quist MJ, Jing X, Lonigro RJ, Brenner JC, Asangani IA, Ateeq B, Chun SY, Siddiqui J, Sam L, Anstett M (2012). The mutational landscape of lethal castration-resistant prostate cancer. Nature.

