# Supplementary material for: Brachyury as a potential modulator of androgen receptor activity and a key player in therapy resistance in prostate cancer
Source: Oncotarget. 2016 Mar 31;7(20):28891–902. doi: 10.18632/oncotarget.8499 (PMC5045364; doi:10.18632/oncotarget.8499)
Supplement: Supplementary file 1 [file oncotarget-07-28891-s001.pdf]

Brachyury as a potential modulator of androgen receptor activity and a key player in therapy resistance in prostate cancer

Supplementary Material

Brachyury as a potential modulator of androgen receptor activity and a key player in therapy resistance in prostate cancer

Filipe Pinto [1,2], Nelma Pértega-Gomes [3], José R Vizcaíno [4], Raquel P Andrade [5,6], Flavio M Cárcano [7,8], Rui M Reis [1,2,8]

Supplementary Table S1. Primer list used in RT-qPCR and in ChIP-qPCR experiments.

Gene	5'-Fw-3'	5'-Rv-3'	Fragment size (bp)	Application
Brachyury	ATGAGCCTCGAATCCACATAGT	TCCTCGTTCTGATAAGCAGTCA	109	RT-qPCR
Plakoglobin	AAGGTGCTATCCGTGTGTCC	GTTGTTGCATGTCAGGTTGG	261	
Snail	CTCTAGGCCCTGGCTGCTAC	TGACATCTGAGTGGGTCTGG	134	
Slug	CTTTTTCTTGCCCTCACTGC	ACAGCAGCCAGATTCCTCAT	161	
E-cadherin	TGCCCAGAAAATGAAAAAGG	GTGTATGTGGCAATGCGTTC	200	
N-cadherin	ACAGTGGCCACCTACAAAGG	TGATCCCTCAGGAACTGTCC	392	
Vimentin	GGGACCTCTACGAGGAGGAG	AAGATTGCAGGGTGTTTTCG	177	
Nestin	CAGGAGAAACAGGGCCTACA	TGGGAGCAAAGATCCAAGAC	243	
CD15	TGGCCCGCTACAAGTTCTAC	CGAGGAAAAGCAGGTACGAG	216	
Musashi	CCGGCTTCGGCCACAGTCTTGGG	GCAGGCAGTAGCGGGTCGGAGTCG	199	
CD 44	AGCAACCAAGAGGCAAGAAA	GTGTGGTTGAAATGGTGCTG	233	
β-actin	GGACTTCGAGCAAGAGATGG	AGCACTGTGTTGGCGTACAG	234	
CD44	GCATTGAACCTCGTGTGAGA	TACCCATGCCCTCAGATTTT	140	ChIP-qPCR
Snail	CTTTGGCTAGGGTGATCAGG	CCGTATCTTCATGACATGGTAGTG	126	
N-cadherin	TCCTCTGTTTTTACAGTTGAAGGA	CAGTGGTAAAAGGGATGCAAA	139	
Fibronectin	TGCCTCAATGAACCTAACTTTTT	AGCCTGCAGGGTATTTTCAC	101	
AMACR	GAACATGAAGGTGCCAGTGA	CCCCAGGAGGAGATGAAAAT	117	
AR	TGGCAACAGTTTTCAGATGTGG	GCAGTCTGACATTCAAACCTGG	103	
β-actin	CCACGATGATGGAACACTTG	CCTCACCCCAACTATGGCTA	199	

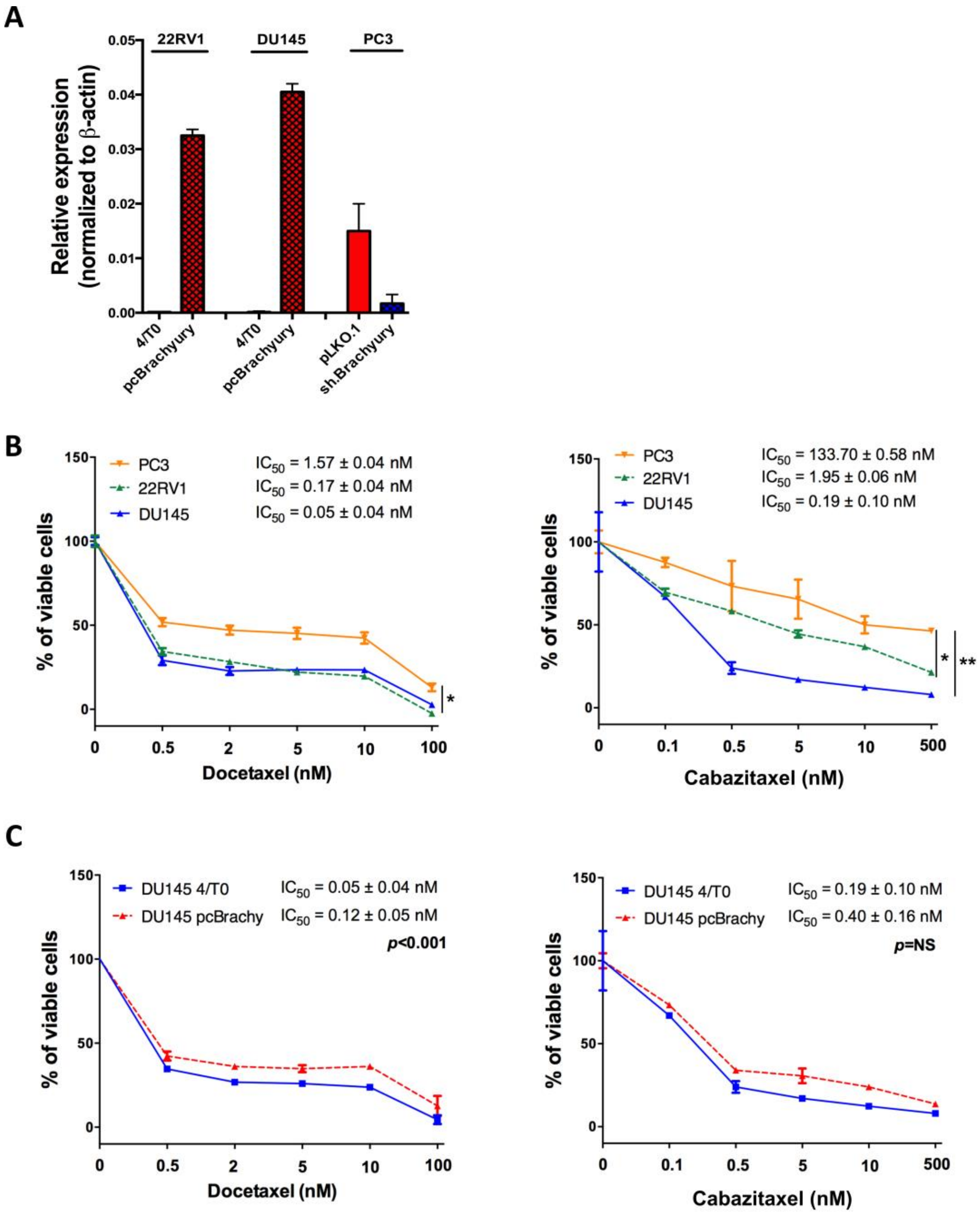

Supplementary Figure S1. Presence of Brachyury is associated with therapy resistance in PCa cells. A) mRNA *Brachyury* levels in transfected 22RV1 and PC3 cells. B) Presence of Brachyury in prostate cancer cell lines is associated with resistance to chemotherapeutic drugs docetaxel and cabazitaxel. C) Brachyury overexpression on DU145 cells increases resistance to therapy. Data are mean ± S.E.M, of 3 biological independent experiments with 3 technical replicates each. NS, non-significative.

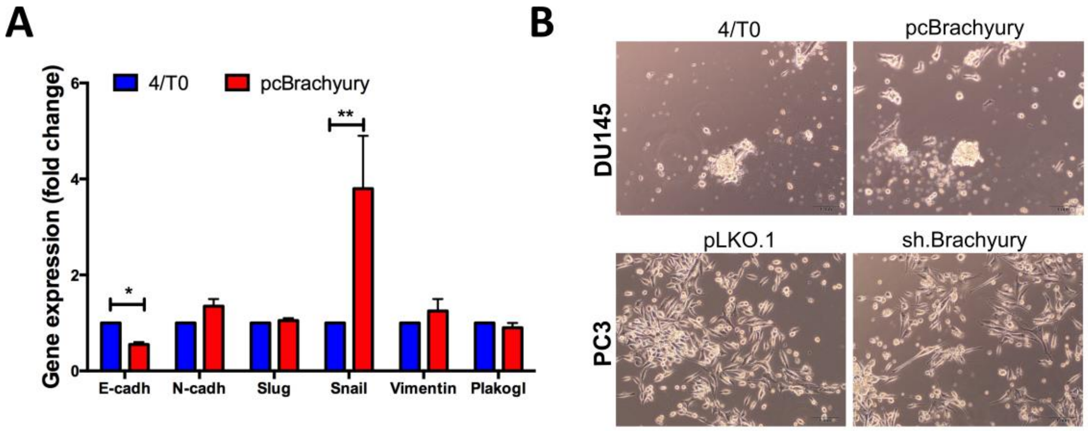

Supplementary Figure S2. Brachyury is associated with EMT in PCa cells. A) In 22RV1 cells Brachyury is able to decrease the expression of epithelial marker *E-cadherin* and increase the expression of mesenchymal marker *Snail*. B). Representative images of DU145 and PC3 cells cultured under stem cell conditions. Du145 and PC3 cells were not able to form prostate-spheres. Data are mean ± S.E.M. of 3 biological independent experiments with 3 technical replicates each. *, $p<0.05$; **, $p<0.01$.

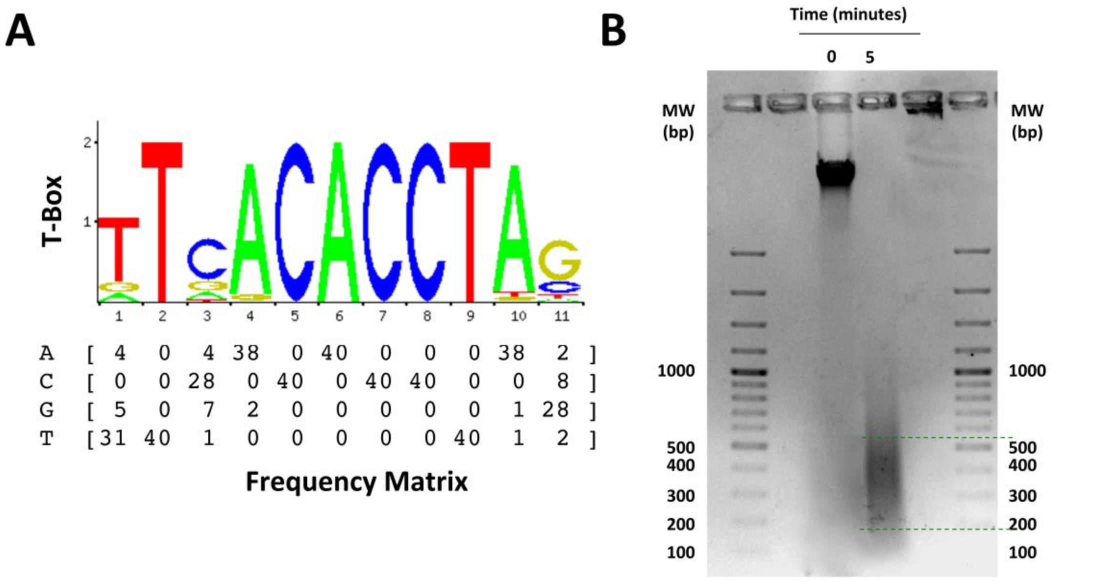

Supplementary Figure S3. Chromatin fragmentation and T-box binding sequence matrix. A) Frequency matrix of the T-Box consensus sequence from JASPAR database. B) Agarose gel for the chromatin before and after to be fragmented for 5 minutes. The bulk of DNA was between 200-500bp.
